# 

**Published:** 1886-11-06

**Authors:** 


### The Hospital.

**An Institution, Family, and Parochial Journal of Hospitals, Asylums, and all Agencies for the Care of the Sick, Criticism and News.**

SATURDAY, NOVEMBER 6TH, 1886.

### BUSINESS COMMUNICATIONS AND ADVERTISEMENTS.

All communications concerning business matters, non-delivery of The Hospital, etc., should be addressed to the Manager, at the Publishing Offices, 30 and 32, Sardinia Street, Lincoln's Inn Fields, London, W.C.

**Cheap Advertisements** of the "Wanted" class, including those for situations, of Twenty-four Words or under, are charged *1s.* an insertion, or three insertions for *2s. 6d.*, but must be paid for in advance. Each additional line of Eight Words is *4d.*

**Employers requiring assistants** are charged for one insertion of Twenty-four Words or under, *2s. 6d.*, or *6s.* for three insertions. Each additional line of Eight Words is *1s.*

**Advertisements** for insertion in the weekly issue of The Hospital **must be delivered** at the Publishing Offices **not later than 11 a.m. on Wednesdays.**

### THE QUILTER PRIZES.

We have the pleasure to announce that we hope to be able to publish the Conditions of the First of the Quilter Prizes, together with the scheme on which they will be offered for competition, in next week's issue.

### NOTES AND NEWS.

### NOTICE TO CORRESPONDENTS.

All correspondence must be written on one side of the paper only.

We cannot undertake to return MSS. not used.

**Communications**, whether intended for insertion or for private information, must be authenticated by the names and addresses of the writers, not necessarily for publication.

**Local papers** containing reports or news paragraphs **should be marked.**

It is especially requested that early intelligence of **local events of interest**, or which it may be thought desirable to bring under notice, may be sent direct to the Editor.

Communications respecting editorial matters should be addressed to the Editor, Norfolk House, Norfolk Street, Strand, London, W.C.

Mr. Henry N. Custance is thanked for his contribution, which will appear in an early number.

West Bromwich District Hospital.—The Secretary of this Institution is Mr. W. H. Laban, not Lehmann, as stated on the 23rd inst.

### THE LITERARY PRIZE.

A Prize of **One Guinea** will be given every fortnight for the best story or incident based upon hospital, or asylum, or student life, or any incident connected with the professions of medicine or nursing. Contributions may relate to any subject mentioned in the Editorial Address published on pp. 1 and 2 of the No. I issue of The Hospital, being the number for 2nd October 1886. The Prize contributions will be published in the first and third numbers of every month, and the prizes duly forwarded to the successful candidates.

### CONDITIONS.

Competitions must be sent in accompanied by the Literary Prize Coupon printed in the advertisement columns of this issue, so as to arrive at the office of The Hospital, Norfolk House, Norfolk Street, Strand, London, W.C., not later than the second and fourth Mondays in each month. Any arriving after these dates will be placed in the following fortnight's competition.

Written competitions must be on one side of the paper only. Printed matter may be sent, and is equally eligible for the Prize. Conciseness, point, and interest will be taken into account in adjudicating the Prizes in these competitions, and any competitor may send in any number of contributions for each competition. The Editor reserves to himself the right to publish any contribution sent in for competition whether successful or not. In the case of printed contributions, the source from which the communication is taken must be stated. Each communication must have the correct name and address of the sender plainly written on the first page, for publication when successful. The omission of these particulars will disqualify. Every contribution must be enclosed in an envelope and have the words "Literary Prize" plainly written in the left-hand corner.

**Results of Second Competition.**

The One Guinea Prize.

No *Story* of sufficient merit having been received, we have decided to award the One Guinea Prize to *Miss M. H. Duncan*, The Cottage Hospital, Blackheath, for her contribution, "In a Hospital Ward," published on p. 57 of The Hospital of Oct. 23rd last.

The Half-Guinea Prize.

We have much pleasure in awarding this prize to the Sub-Dean, Belmont, Parkstone, Dorset, for his excellent contribution of anecdotes, which we propose to publish in an early number.

The Five-Shilling Prize.

Our readers do not seem to understand that this prize will be given for the best *Item of News* for the "Notes and News" column. At least that is the only way in which we can account for the absence of competitors for this prize during the last fortnight. Anyone who reads the "Notes and News" should have no difficulty in contributing to this column, and so entering for the prize.

### "THE HOSPITAL" CHARITY BOX.

Our "Charity Box" is intended to afford a little practical help to the hospitals. Each week we propose a word for "anatomical dissection," and the first 150 competitors who extract the largest number of words from it receive the following prizes:—

| | |
|---|---|
| 1st .. .. .. | One Guinea. |
| 2nd .. .. .. | Half a Guinea. |
| 3rd .. .. .. | Five Shillings. |

The above Prizes will be repeated for every additional 150 competitors who may enter each week.

Sixth Competition.

Compose the largest number of words you can from "CHARITY BOX."

*Observe:* Proper names, abbreviations, foreign and obsolete words, and repetitions are barred.

Write only on one side of the paper, and total your words.

Append your real name and address, adding whether Mr., Mrs., or Miss, etc.

All replies, addressed "Charity Box," The Hospital, Norfolk House, Norfolk Street, W.C., must arrive by Friday, November 12th. Results will appear Saturday, Nov. 20th.

*Every Competitor is required to cut out and enclose with his list the "Charity Box" Coupon* (see advertisement pages), *and a shilling entrance fee* (postal orders only).

The proceeds resulting from each "Charity Box" competition will be funded until the end of December 1886. The total sum available for distribution, and the names of the hospitals selected for donations, will appear on Saturday, January 8, 1887. The distribution of the proceeds will be at the Editor's sole discretion.

Note.—The words "Charity" and "Box," do *not* count in this competition.

Result of Fourth Competition.

The "Sydenham" Competition has resulted as follows:—

| | Score. | |
|---|---|---|
| Miss Coker, 7, Saxe Weimar Terrace, Southsea .. .. .. .. .. .. .. .. | 210 | 1st Prize. |
| Mr. James High, chief attendant, R.N. Hospital, Great Yarmouth .. .. .. .. .. | 200 | 2nd Prize. |
| Miss Louie Pepper, 37, Bethel St., Norwich .. | 195 | 3rd Prize. |

We are glad to see that correspondents are endeavouring to comply with our rules.

In view of the object which we have in view, we should be glad to see a much more general response made to the "Charity Box." This week, for instance, the replies have numbered only 30, which represents an amount which does not cover the prizes. The number ought easily to be increased tenfold.

The prizes have been forwarded to the winners.

### THE CHILDREN'S CORNER.

"You know," says Diosota, "I am afraid some of your puzzles sorely tax your little guessers, for they are so difficult—at least some of them are." Diosota herself answers so well, that I am forced to fancy that she writes on behalf of others rather than of herself. When puzzles are too easy, there is not much fun in guessing, and, moreover, a fairly difficult puzzle exercises the thinking faculty. If little children were more generally trained to *think*, the world would not be so full as it is of mere automata.

### OUR PASTIMES.

**Children's Quarterly Prize Competition.**

| | |
|---|---|
| Began .. .. .. .. | 2nd Oct., 1886 |
| Ends .. .. .. .. | 25th Dec., 1886 |

PRIZES.

| | |
|---|---|
| 1st .. .. .. .. | Thirty Shillings |
| 2nd .. .. .. .. | Twenty " |
| 3rd .. .. .. .. | Ten " |
| 4th .. .. .. .. | Five " |

will be awarded to the four competitors, who shall have sent in, during the thirteen weeks, the largest number of correct answers to our pastimes.

**Sixth Competition.**

No. 27. What nation resembles Mr. Bass, the famous brewer?

No. 28. Who can translate "Equus est in stabulo et non est?"

No. 29. Why is "s" the most terrible letter in the alphabet?

No. 30. Turn *boot* into *shoe* in the same way as indicated on page 66.

No. 31. The following letters were discovered on the wall of an old country church. It was observed that the addition of a particular letter would at once turn the whole into a sentence. What is the letter?

R.M.M.B.R.M.Y.P.R.F.C.T.M.N.
V.R.K.P.T.H.S.P.R.C.P.T.S.T.N.

No. 32. A milkman had an 8 pint can full of milk; and a 5 pint and a 3 pint can, the two latter being empty. He wanted to measure off 4 pints. How did he do it?

**Results of Fourth Competition.**

No. 16. "Foe" spelt without any of its letters is "N-M-I" (enemy). Two of my little readers have discovered the answer. "Mikado" ingeniously suggests "Sin."

No. 17. This proved an easy puzzle, for everyone has guessed "Hospital."

No. 18. "Little Mary" is positive that the longest English word must have more than six letters, but she adds, "there must be some catch I cannot see." "Smiles" is the longest word we have in English, since there is a "mile" between the first and last letters.

No. 19. I did not think this word would be put into shape so easily as it has been. The word is "Senselessness."

No. 20. My little friends have exercised much ingenuity over this. "Adam" has been most generally given as the "first man" referred to in the Bible, but puzzles, you know, require crooked answers, and the by-paths must therefore be sought out. Florrie suggests King James, but his dread majesty's name occurs in the dedication which precedes the Bible, rather than in the Bible itself. T. K. alone comes off victor. My little friends, look at the top of the chapter which commences Genesis, and you will find that the first man was Chap. I!

I notice one or two little correspondents have not answered this week, but I hope to hear from them next.

All right—None.
Four right—T. K., Ethel, Filomel, Retsibsi, Diosota.
Three right—Mikado, Little Mary, Hetty.
Two right—Florrie, Alsie, Ubique, Bismarck.
One right—C. Tynget.

Answers, having the actual name and address (to which a pseudonym may be added for publication), must have "The Children's" Coupon attached (see advertisement pages), addressed to the "Puzzle Editor, The Hospital, Norfolk House, Norfolk Street, Strand, W.C.," not later than Thursday, 11th November. Results will appear in our issue of the 20th November.

### The In- and Out-Patients' Hospital Diary.

The following forms the first instalment of our combined In- and Out-Patients' Diary, and comprises twenty-three General, the Cancer and Children's Hospitals in the Metropolis. The Diary will be continued week by week, so as to be completed in each monthly part.

### I.—GENERAL HOSPITALS.

| Hospitals and Terms of Admission. | Physicians, Surgeons, and Medical Officers, with Days and Hours of Attendance. |
|---|---|
| Charing Cross Hospital, West Strand, W.C.<br>Cases of accident or emergency are immediately admitted as in-patients. Subscriber's letter expected in other cases<br>Out-patients admitted without subscribers' letters first time; for continued attendance they must be obtained from the Secretary | *In-Physicians*, Drs. Pollock, M. Th.; Green, W. S.; Bruce, Tu. F. Hour, 1.30<br>*In-Surgeons*, Messrs. Barwell, M. Th.; Bellamy, Tu. F.; Bloxam, W. S. Hour, 1.30<br>*Out-Physicians*, Drs. Wilcocks, M.Th.; Abercrombie, Tu. F.; Lubbock, W. S.; Murray, W. S. Hour 1.30<br>*Out-Surgeons*, Messrs. Cantlie, M.Th.: J. H. Morgan, Tu.F.; Stanley Boyd, W. S. Hour, 1.30 |
| French Hospital, 10, Leicester Place, Leicester Sq., W.C.<br>Free to all foreigners speaking French | *In- and Out-Physician*, Dr. Vintras, Tu. F. 10 to 11 a.m.<br>*In- and Out-Surgeons*, Messrs. H. de Méric, M. Th. 10; J. Késer, W. S. 10 |
| German Hospital, 113, Dalston Lane, E.<br>Free, or by Governor's letter. Urgent cases free at all times | *In-Physicians*, Drs. Weber, M. T. 2;<br>*In-Surgeons*, Drs. Lichtenberg and Burger, W. S. 2<br>*Out-Physicians*, Drs. Tuchmann, Tu. W. 2; Ludwig, M. Th. 2; Keller, Tu. W. F. 2; Reihlen, M. Th. F. 2 (*Men*, Tu. Th.; *Women*, M. W. F.) |
| ——— Eastern Branch, 49, Mansell Street, Aldgate, E. | *Out-Physician*, Dr. C. Harrer, Tu. F. 8 to 10 a.m. |
| ——— Western Branch, 259, Oxford Street, W. | *Out-Physician*, Dr. M. Castaneda, M. T. 8 to 10 a.m. |
| Great Northern Central Hospital, Caledonian Road, N.<br>Free to all, without letters of recommendation | *In-Physicians*, Drs. Cholmeley, Cook, and Burnett, M. Tu. Th. 2, S. 10<br>*In-Surgeons*, Messrs. W. Adams, W. Watson, and J. Macready, M. Tu. Th. F. 2<br>*Out-Physicians*, Drs. Beale, M. Th. 2; Beevor, Tu. 2, S. 10<br>*Out-Surgeons*, Messrs. Lockwood, M. Th. 2; Makins, Tu. F. 2 |
| Guy's Hospital, St. Thomas's Street, S.E.<br>Admission free, without letters of recommendation | *In-Physicians*, Drs. Pavy, Tu. F.; Pye-Smith, M. Th. S.; Taylor, M. Tu. Th. F. Hour, 1.30<br>*In-Surgeons*, Messrs. Bryant, M. Th. Durham, M. Th. F.; Howse, W. S.; D. Colley, M. Th. Hour, 1.30<br>*Out-Physicians*, Drs. Goodhart, F.; Carrington, W.; Hale White, M. Hour, 12.30<br>*Out-Surgeons*, Messrs. Lucas, Th.; Golding Bird, S.; Jacobson, W.; Symonds, M. Hour, 12.30 |
| King's College Hospital, Portugal Street, W.C.<br>By Governors' letters; accidents and urgent cases free at all times | *In-Physicians*, Drs. Beale, Duffin, Yeo, Tu. W. F. 1.30; Tu. W. F. S. 2<br>*In-Surgeons*, Mr. Wood, Sir J. Lister, Messrs. H. Smith, Cheyne, W. Rose, Barrow. Daily, 1.30<br>*Out-Physicians*, Drs. Ferrier, J. Curnow, N. I. C. Tirard; *Men*, Tu.Th.; S. 1; *Women*, M. W. F. 1<br>*Out-Surgeons*, Messrs. W. Rose, W. Cheyne; *Men*, Tu. Th. S. 1; *Women*, M. W. F. 1 |
| London Hospital, Whitechapel Road, E.<br>Admission free. Out-patients are required to attend only once, unless otherwise directed by the Physician or Surgeon | *In-Physicians*, Drs. Mackenzie, Tu. F.; Down, Tu. F.; H. Jackson, M. Th.; Sutton, M. Th.; Fenwick, Tu. F. Hour, 1.30<br>*In-Surgeons*, Messrs. Rivington, Tu. F.; Couper, Waren Tay, M. Th.; McCarthy, W. S.; Treves, Tu. F. Hour, 1.30<br>*Out-Physicians*, Drs. Sansom, Th.; M. Ralfe, M. Th.; Turner, Tu. F.; Warner, Tu. F.; Gilbart Smith, W. S. J. Anderson, W. S. Hour, 1.30<br>*Out-Surgeons*, Messrs. Mansell-Moullin, M. Th.; Eve, M. W.: Reeves, Tu. S.; H. Fenwick, Tu. F. Hour, 1.30 |
| London Temperance Hospital, Hampstead Road, N.W.<br>For treatment without alcohol | *In- and Out-Physicians*, Drs. J. Edmunds, R. J. Lee. 1.30 to 2<br>*In- and Out-Surgeons*, Messrs. A. P. Gould, G. C. Wilkin, M. Tu. Th. S. 1.30 to 2 |

| Hospitals and Terms of Admission. | Physicians, Surgeons, and Medical Officers, with Days and Hours of Attendance. |
|---|---|
| Metropolitan Free Hospital, Kingsland Road, N.<br>Admission free | *In- and Out-Physicians*, Drs. Drysdale, Tu. F. 12; J. G. Dudley, W. S. 12; Fotherby, M. Th. 12; H. H. Tooth, Tu. F. 9; A. Haig, W. S. 9; A. Davies, M. Th. 9<br>*In- and Out-Surgeons*, Messrs. E. J. Chance, W. S. 9; Goodsall, Tu. F. 9; Walsham, M. Th. 9; A. A. Bowlby, F. 9; S. Paget, Th. 9 |
| Middlesex Hospital, Berners Street, W.<br>Admission by ticket. Accidents and urgent cases are admitted free | *In-Physicians*, Drs. Cayley, M. Th.; Coupland, Tu. F.; D. Powell, M.Th.; Finlay, Tu. F. Hour, 1.30<br>*In-Surgeons*, Messrs. Hulke, M. Th.; Lawson, M. Th.; Morris, Tu. F. Hour, 1.30<br>*Out-Physicians*, Drs. Fowler, Tu. F. 3.30; Biss, M. Th. 1.30; Pringle, W. F. S. 1.30<br>*Out-Surgeons*, Messrs. Andrew Clark, M. Tu. 1.15; Pearce Gould, Tu. F. 1.30; J. B. Sutton, W. S. 9 |
| North-West London Hospital, 18, Kentish Town Road, N.W.<br>By subscribers' free tickets | *In and Out-Physicians*, Drs. W. C. Hood, J. Shaw, Edwardes, Wolfenden. Daily, 1.30<br>*In and Out-Surgeons*, Messrs. F. Durham, Collier, Jennings, J. Black. Daily, 1.30 |
| Ospedale Italiano, 41, Queen Square, Bloomsbury<br>Admission free to persons of all nationalities, but Italians have preference | *In- and Out-Physicians*, Drs. M. Castaneda, M. W. 11.30 to 1; Tu. F. 10 to 11, J. W. B. Steggall, W. 10 to 11, S. 4 to 5 |
| Poplar Hospital, 303, East India Dock Road, E.<br>Free to accidents and emergencies | *In-Surgeons*, Messrs. F. M. Corner, M. Brownfield, and Resident Surgeons<br>*Out-Physician*, Dr. J. D. Grant, W. S. 12 to 2<br>*Out-Surgeon*, Mr. T. E. Bowkett, Tu. F. 12 to 2 |
| Royal Free Hospital, Gray's Inn Road, W.C.<br>Admission free | *In- and Out-Physicians*, Drs. Cockle, M. Th.; Sainsbury, T. F.; West, W. S. Hour, 1<br>*In- and Out-Surgeons*, Messrs. Rose, M. T.; W. Gant, Tu. F.; Brow, W. S. Hour, 1 |
| St. Bartholomew's Hospital, Smithfield.<br>Admission free, without letter or ticket | *In-Physicians*, Drs. Andrew. Church, Gee, Sir D. Duckworth, N. Moore. Daily, 11 and 1.30<br>*In-Surgeons*, Messrs. Savory, T. Smith, Willett. Langton, M. Baker. Daily, 12.30 and 1.30<br>*Out-Physicians*, Drs. Hensley, M. Th.; Brunton, Tu. F.; Legg, W. S. Hour, 11<br>*Out-Surgeons*, Messrs. Marsh, Tu. F.; Butlin, M. Th.; Walsham, W. S. Hour, 12 |
| St. George's Hospital, Hyde Park Corner, S.W.<br>By Governor's letter. Free to accidents and emergencies. Vaccination on Th. at 11 | *In-Physicians*, Drs. Wadham, M. F.; Dickinson, M. Tu. Th. F.; Whipham, Tu.W. S.; Cavafy, Tu. S. Hour, 1<br>*In-Surgeons*, Messrs. Holmes, M. Th. F. 1, W. 1.30: Rouse, M. Th. F. 1, W. 1.30; Haward, Tu. Th. S. 1, W. 1.30<br>*Out-Physicians*, Drs. Ewart, M. F. 10.30 to 12; Owen, Tu. S. 10.30 to 12<br>*Out-Surgeons*, Messrs. Bennett, M. F. 10.30 to 12: Dent, Tu. S. 10.30 to 12 *Men*, F. S.; *Women*, M. Tu. |
| St. Mary's Hospital, Cambridge Place, Paddington, W.<br>By Governor's letter | *In-Physicians*, Drs. E. Sieveking, Tu. F. 1.45; Broadbent, M. Th. 1.45; Cheadle, W. 1.45, S. 9.30<br>*In-Surgeons*, Messrs. Norton, M. Th. 1.45; Owen, Tu. F. 1.45; H. Page. W. S. 9.30<br>*Out-Physicians*, Drs. D. B. Lees, W. S.; S. Phillips, M. Th; R. Macguire, Tu. F. Hour, 1.30<br>*Out-Surgeons*, Messrs. W. Pye, M. Th.; A. J. Pepper, W. S. Hour, 1.30 |

### GENERAL HOSPITALS.—*continued.*

| Hospitals and Terms of Admission. | Physicians, Surgeons, and Medical Officers, with Days and Hours of Attendance. |
|---|---|
| St. Thomas's Hospital, Westminster Bridge Road, S.E. By Governor's letter. Accidents and emergencies free at all times. Vaccination on Wednesday at 11.30 | *In-Physicians*, Drs. Bristowe, Tu. F.; Stone, M. Th.; Ord, M. Th.; J. Harley, Tu. F.; Gervis, Tu. F. Hour, 2<br>*In-Surgeons*. Messrs. Sydney Jones, Tu. F.; Croft, M. Th.; Sir W. MacCormac, M. Th. Hour, 2<br>*Out-Physicians*, Drs. Payne, Tu. F; Sharkey, M. Th.; Gulliver, W. S. Hour, 1.30<br>*Out-Surgeons*, Messrs. MacKellar, M. Th.; Clutton, Tu. F.; Anderson, W. S.; Pitts, M. Tu. Hour, 1.30 |
| Seamen's Hospital (late Dreadnought), Greenwich, S.E. Entirely free to seamen, and to all accident cases | *In- and Out-Physicians*, Dr. F. Curnow and H. Griffiths.<br>*In- and Out-Surgeons*, Messrs. W. J. Smith; G. R. Turner. Daily, between 9 and 3 |
| Tottenham Training Hospital, Tottenham, N. | *In-Physician*. Dr. Rasch, Tu. F. 3 to 5<br>*In-Surgeons*, Dr. Lichtenberg, M. Th. 3 to 5; Mr. E. H. May, M. Th. 3 to 5<br>*Out-Surgeon*, Dr. Lloyd Smith, M. W. 11 to 2; *Men* only, W. 7 to 8 p.m. |
| University College Hospital, Gower Street, W.C. By Governors' letters. Accidents and emergencies free | *In-Physicians*, Drs. Wilson Fox, Tu. Th. 2, S. 9.30; Ringer, M. W. F. 1.30; Bastian, M. 2.30, W. 10, F. 2.30; F. Roberts, Tu. Th. 1.15, S. 9.30<br>*In-Surgeons*, Messrs. B. Hill, Tu. F. 2.15; C. Heath, M. W. F. 2; M. Beck, Tu. Th. S. 2<br>*Out-Physicians*, Drs. Gowers, W. S. 1.30; Poore, Tu. F. 1.30; Barlow, M. Th. 1.30<br>*Out-Surgeons*, Messrs. Barker, M. Th. 1.30; Godlee, Tu. F. 1.30; Horslee, W. S. 1.30 |
| West London Hospital, Hammersmith Road, W. By Governors' letters. New cases must attend at 1.30 | *In-Physicians*, Drs. G. Rogers, D. W. Hood, F. Drewitt<br>*In-Surgeons*, Messrs. C. Keetley, F. Edwards, W. B. Clarke<br>*Out-Physicians*, Drs. Herringham, M. Th.; Savill, Tu. F.; Ball, W. S. Hour, 2<br>*Out-Surgeons*, Messrs. Ballance, Tu. F.; Weiss, M. Th.; Wainewright, W. S. Hour, 2 |
| Westminster Hospital, Broad Sanctuary, S.W. By Governors' letters. Accidents and urgent cases free | *In-Physicians*, Drs. Sturges, M. Th. S.; Allchin, M. Tu. F.; Donkin, Tu. W. S. Hour, 1.30<br>*In-Surgeons*, Messrs. Cowell, M. Tu. Th.; Davy, M. Tu. F.; Macnamara, M. W. S. Hour, 1.30<br>*Out-Physicians*, Drs. De H. Hall, M. Th.; A. H. Bennett, Tu. F.; Murrell, W. S. Hour, 1.30<br>*Out-Surgeons*, Messrs. Cooke, M. Th.; Bond, Tu. F.; Barrow, W. S. Hour, 1.30 |

### II.—SPECIAL HOSPITALS.

### CANCER.

| | |
|---|---|
| Cancer Hospital, Brompton, S.W. Entirely free | *In- and Out-Surgeons*, Drs. A. Marsden, H. Snow, M.; F. A. Purcell, F.; Messrs. F. B. Jesset, W.; W. Elam, S.; C. Stonham, Th.; C. Jennings, Tu. Hour, 2 |
| London Hospital, Whitechapel Road, E. Free | Daily, 1.30. See *Surgeons*, p. 103 |
| Middlesex Hospital, Berners Street, W. Free. See p. 103. | *In- and Out-Surgeon*, Mr. Morris, Th. 1.30 |
| St. Saviour's Hospital, Osnaburg Street, N.W. By Governors' letters. For the reception of persons who wish to be treated by the medicines of Count Mattei | *In- and Out-Physician*, Dr. A. Kennedy, 10.30 and 4 daily |

### CHILDREN'S DISEASES.

| | |
|---|---|
| Alexandra Hospital for Children, 18, Queen's Sq., W.C. No out-patients. For hip disease | *In-Surgeons*, Messrs. H. Marsh and A. Bowlby |

### CHILDREN'S DISEASES.—*continued.*

| Hospitals and Terms of Admission. | Physicians, Surgeons, and Medical Officers, with Days and Hours of Attendance. |
|---|---|
| All Saints' Children's Hospital, 4, Margaret Street, W. For chronic joint diseases. Children under 10 | *In-Surgeon*, Mr. N. Smith |
| Belgrave Hospital for Children, 79, Gloucester Street, Pimlico. By Governors' letters | *In- and Out-Physicians*, Drs. W. Hope and W. Ewart, M. Tu. Th. F. 9<br>*In- and Out-Surgeons*, Messrs. C. T. Dent and Margerison, M. Tu. Th. F. 9 |
| Cheyne Hospital for Sick and Incurable Children, 46, Cheyne Walk, Chelsea, S.W. In-patients only | *In-Physician*, Dr. G. V. Poore<br>*In-Surgeons*, Messrs. J. Macready and J. Bartlett |
| East London Hospital for Children and Dispensary for Women, Glamis Road, Shadwell, E. By Governor's letter. Urgent accident cases free | *In- and Out-Physicians*, Drs. E. Smith, H. Donkin, H. Crocker, J. A. Coutts, and D. Williams, M. Tu. W. Th. F. 1 to 1.30 (new cases); 3 to 3.30 (old cases)<br>*In- and Out-Surgeons*, Messrs. A. Cæsar, H. A. Reeves, R. Parker, L. A. Dunn, Tu. F. 9 |
| Evelina Hospital for Sick Children, Southwark Bridge Road, S.E. Free, but Governor's letter takes precedence | *In-Physicians*, Drs. F. Taylor and J. Goodhart<br>*In-Surgeons*, Messrs. H. Howse and R. C. Lucas<br>*Out-Physicians*, Drs. N. Tirard and F. Wilcocks. Daily, 9<br>*Out-Surgeons*, Messrs. C. Symonds and G. Makins. Daily, 9 |
| German Hospital, Dalston Lane, E. | *In- and Out-Physician*, Dr. Rasch, M. Th. 9 |
| Great Northern Central Hospital. | W. 2.30. See p. 103 |
| Grosvenor Hospital for Women and Children, Vincent Sq., Westminster, S.W. By Subscriber's letter or payment | *In- and Out-Physicians*, Drs. A. Smythe, R. Gibbons, and Steavenson, M. Tu. Th. S. 2 to 3<br>*In- and Out-Surgeon*, Mr. Parker, M. Tu. Th. S. 2 to 3 |
| Hospital for Sick Children, 49, Great Ormond St., W.C. By Governors' letters. Other cases investigated. Patients under 12 | *In-Physicians*, Drs. W. Cheadle, Tu. F.; Sturges, W. S.; Barlow, M. Th. Hours, 9 to 11<br>*In-Surgeons*, Messrs. H. Marsh, W. S.; E. Owen, W. S. Hours, 9 to 11<br>*Out-Physicians*, Drs. R. J. Lee and Abercrombie, M. Th.; Lubbock and Money, T. F. Hours, 8.30 to 10<br>*Out-Surgeons*, Messrs. Morgan and Pitts, W. S. 8.30 to 10 |
| King's College Hospital. By Governor's letter. See p. 16 | *In- and Out-Physician*, Dr. T. C. Hayes, W. F. 1 |
| North Eastern Hospital for Children, Hackney Road, E. By subscriber's free ticket; or by payment of 4*d*. on admission, and 3*d*. per week | *In- and Out-Physicians*, Drs. W. Cayley, F. Turner, C. Semple, and W. Pasteur, M. W. Th. S. 1.30<br>*In- and Out-Surgeons*, Messrs. Warren Tay and Godlee, M. 1.30, F. 9 |
| Paddington Green Children's Hospital, Paddington Green, W. Admission free | *In- and Out-Physicians*, Drs. T. Brunton, J. Ogilvie, G. Laycock, and S. Phillips. Daily, 9.15<br>*In- and Out-Surgeons*, Messrs. W. Cheyne and S. Boyd. Daily, 9 |
| Royal Hospital for Children and Women, Waterloo Bridge Road, S.E. Out-Patients pay 1*d*. on each attendance. Boys (out-patients) not admissible over 12; (in-patients) over 8 | *In-Physicians*, Drs. Park, M. Th.; Gulliver, Tu. F.; Price, W. S. Hour, 2<br>*In-Surgeon*, Mr. W. H. A. Jacobson, M. Th.<br>*Out-Physicians*, Drs. Park, M. Th., Dakin, W. F.; Hadden, Tu. S. Hour, 2<br>*Out-Surgeon*, Mr. E. O. Day, W. 2 |
| St. Mary's Hospital, Paddington. Children over 2 | M. Th. 1.30. See p. 103 |
| St. Thomas's Hospital, Westminster Bridge Road | *In- and Out-Physician*, Dr. Cory, W S. 1.30. See p. 103 |
| Samaritan Free Hospital for Women and Children, Lower Seymour Street, W.; Branch, 1, Dorset St., Manchester Sq., W. Governor's letter or card is necessary if the disease of the applicant is *not* peculiar to her sex | *In- and Out-Physicians*, Drs. M. Prickett, A. Routh, and W. Griffiths. Daily, 12 to 2<br>*In- and Out-Surgeons*, Messrs. Meredith, A. Doran, and J. Malcolm. Daily, 12 to 2 |

